# A high-throughput microfluidic device based on controlled incremental filtration to enable centrifugation-free, low extracorporeal volume leukapheresis

**DOI:** 10.1038/s41598-022-16748-5

**Published:** 2022-08-13

**Authors:** Dalia L. Lezzar, Fong W. Lam, Ravin Huerta, Anton Mukhamedshin, Madeleine Lu, Sergey S. Shevkoplyas

**Affiliations:** 1 Department of Biomedical Engineering, University of Houston, 3605 Cullen Blvd, Houston, TX 77204-5060 USA; 2 Division of Pediatric Critical Care Medicine, Baylor College of Medicine, Houston, TX 77030 USA

**Keywords:** Biomedical engineering, Paediatrics, Therapeutics

## Abstract

Leukapheresis, the extracorporeal separation of white blood cells (WBCs) from red blood cells (RBCs) and platelets (PLTs), is a life-saving procedure used for treating patients with cancer and other conditions, and as the initial step in the manufacturing of cellular and gene-based therapies. Well-tolerated by adults, leukapheresis poses a significant risk to neonates and low-weight infants because the extracorporeal volume (ECV) of standard centrifugation-based machines represents a particularly large fraction of these patients’ total blood volume. Here we describe a novel high-throughput microfluidic device (with a void volume of 0.4 mL) based on controlled incremental filtration (CIF) technology that could replace centrifugation for performing leukapheresis. The CIF device was tested extensively using whole blood from healthy volunteers at multiple hematocrits (5–30%) and flow rates (10–30 mL/min). In the flow-through regime, the CIF device separated WBCs with > 85% efficiency and 10–15% loss of RBCs and PLTs while processing whole blood diluted with saline to 10% hematocrit at a flow rate of 10 mL/min. In the recirculation regime, the CIF device demonstrated a similar level of separation performance, virtually depleting WBCs in the recirculating blood (~ 98% reduction) by the end of a 3.5-hour﻿ simulated leukapheresis procedure. Importantly, the device operated without clogging or decline in separation performance, with minimal activation of WBCs and PLTs and no measurable damage to RBCs. Compared to the typical parameters of centrifugation-based leukapheresis, the CIF device had a void volume at least 100-fold smaller, removed WBCs about twice as fast, and lost ~ 2–3-fold fewer PLTs, while operating at a flow rate compatible with the current practice. The hematocrit and flow rate at which the CIF device operated were significantly higher than previously published for other microfluidic cell separation methods. Finally, this study is the first to demonstrate a highly efficient separation of cells from recirculating blood using a microfluidic device. Overall, these findings suggest the feasibility of using high-throughput microfluidic cell separation technology to ultimately enable centrifugation-free, low-ECV leukapheresis. Such a capability would be particularly useful in young children, a vulnerable group of patients who are currently underserved.

## Introduction

Leukapheresis is a complex medical procedure during which a patient’s blood is passed through an apheresis machine to collect white blood cells (WBCs) and return red blood cells (RBCs) and platelets (PLTs) back to the patient^1,2^. Leukapheresis enables two potentially life-saving applications: leukodepletion and WBC collection. Leukodepletion can be used to reduce a dangerously high WBC count in patients with leukemia^3,4^, or to remove activated WBCs as a drug-free treatment for inflammatory bowel disease^5–7^ and other conditions^8,9^. Collection of WBCs via leukapheresis is the initial step in manufacturing a wide range of cellular therapies^2^, including granulocyte infusion^10,11^, adoptive immunotherapies^12–15^, hematopoietic stem cell transplantation^16–18^, and novel gene-based treatments^19,20^.

Although generally well-tolerated by adults and older children, performing leukapheresis for neonates and low-weight infants is technically challenging and clinically risky. Currently, leukapheresis is performed using centrifugation-based apheresis machines, which have a substantial extracorporeal volume (ECV) typically ranging 150–250 mL (excluding any additional tubing to connect to the patient)^2,21^, whereas total blood volume (TBV) of a 10 kg infant is only ~ 750 mL^22,23^. Because the ECV represents such a large fraction of their TBV, pediatric patients experience a significantly higher incidence of serious complications associated with the leukapheresis procedure, including hypotension, symptomatic hypocalcemia, allergic reactions, catheter-related thrombosis, infections, severe anemia, and even death^21,24–28^. In principle, WBCs can also be separated using a regular leukoreduction filter^29,30^, or a column packed with cellulose acetate beads that selectively adsorb activated granulocytes and monocytes^6^. However, the separation capacity of these devices is limited by their physical size, WBCs trapped by the devices continue to release proinflammatory cytokines into the bloodstream, and the recovery of trapped WBCs via backflush is rather modest^6,29,30^.

The earliest attempt to miniaturize leukapheresis using microfluidics utilized a continuous-flow diffusive filter to remove WBCs from a sample of blood with high efficiency (~ 97%), albeit with a significant RBC loss (~ 50%) and a flow rate of only 5 µL/min^31^. Since then, the use of microfluidic technologies for blood cell separation (although not specifically for leukapheresis) has steadily increased^32^. Devices based on ‘inertial focusing’^33,34^ can perform cell separation at relatively high flow rates (1–20 mL/min) but require significant dilution of the blood sample (< 1% hematocrit, HCT)^35–37^. Devices based on ‘deterministic lateral displacement’ (DLD)^38^ require significantly less dilution, but have exceedingly small minimal feature sizes (< 5 µm), require multiple pumps and extremely high driving pressures to operate at reasonable flow rates (< 7 mL/min), and often subject cells to shear stress sufficiently high to activate PLT and von Willebrand factor^39–42^. ‘Controlled incremental filtration’ (CIF) overcame these limitations by having a substantially larger minimal feature size (~ 20 µm), and thus reducing fluidic resistance and shear throughout the device^43,44^. As a result, a CIF-based device was able to separate > 85% of WBCs (with < 30% loss of RBCs and PLTs) from the mononuclear cell (MNC) concentrates (~ 5% HCT) at flow rates of up to 30 mL/min^46^ and PLTs concentrated and leukoreduced using CIF were minimally activated^43,44^. Notwithstanding these significant advancements, none of the aforementioned devices have been tested in the recirculation regime for any significant amount of time, and the effects of such processing on blood cells and device performance remain largely unknown.

In this study, we describe a new CIF-based device with a void volume of only 0.4 mL designed for high-throughput separation of WBCs from minimally diluted, recirculating whole blood. We first tested the separation performance of the device in a flow-through regime while adjusting multiple variables independently: HCT (5–30%), filtrate:retentate flow ratio (2–13:1), and flow rate (10–30 mL/min). The device demonstrated > 85% efficiency of WBC removal (with only 10–15% loss of RBCs and PLTs) while processing diluted whole blood with 10% HCT at a flow rate of 10 mL/min. When tested in the recirculation regime, the device operated without clogging or any decline in separation performance, with minimal activation of WBCs and PLTs and no measurable damage to RBCs over the entire duration of a 3.5-hour﻿ simulated leukapheresis procedure. The WBC concentration in the recirculating blood declined exponentially, decreasing ~ 98% by the end of the procedure.

## Results

### Design and operation of the microfluidic device

**Figure 1:**
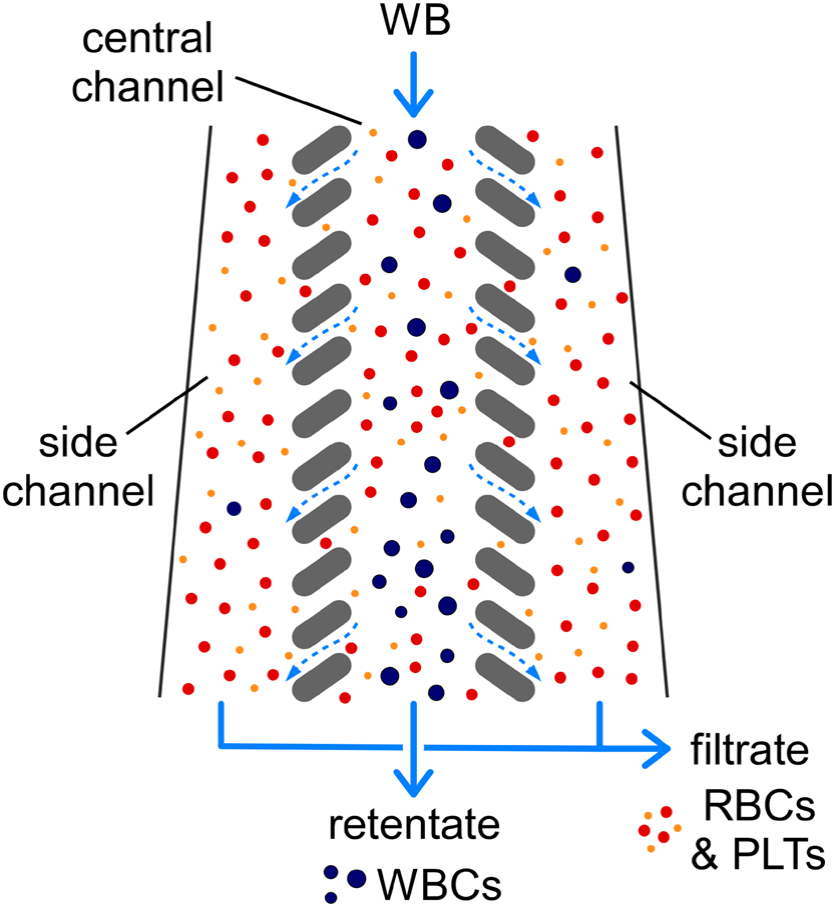
The separation of WBCs from RBCs and PLTs via controlled incremental filtration (CIF). Most WBCs are too large to be pulled by the filtrate into the side channels, and therefore they are retained and concentrated in the central channel. RBCs and PLTs are smaller than the critical diameter of the device, and therefore they are distributed between the filtrate and the retentate according to the device flow ratio.

The operating principle of controlled incremental filtration (CIF) and the computational framework used for generating CIF-based device designs have been described in detail previously^43,44^. A typical CIF design consists of three collinear flow channels separated by a series of ‘pill’-shaped posts, which define the filtration gaps connecting the central and the side channels fluidically (Fig. 1). As the blood sample flows through the central channel, a small fraction of that flow is siphoned off into the side channels through each filtration gap. The width of the side channels gradually increases to accommodate the influx of filtrate. The rate of this increase is calculated, as previously described^43^, to precisely control the fraction of flow extracted through each gap. It is the magnitude of this flow fraction—not the width of the gaps—that determines the size cutoff for the cells that are too large to be pulled into the side channels by the filtrate (‘critical diameter’). The critical diameter of the CIF design used in this study was 6 µm to retain most WBCs in the central channel while allowing RBCs and PLTs to outflow into the side channels unimpeded (Fig. 1). Because the flow fraction extracted through each gap is relatively small, any CIF design must incorporate thousands of gaps to achieve the desired filtrate:retentate flow ratio^43,44^. In practice, the number of filtration gaps (and, hence, the flow ratio) is limited by the maximum channel length allowed by the fabrication technique^44,46^.

**Figure 2:**
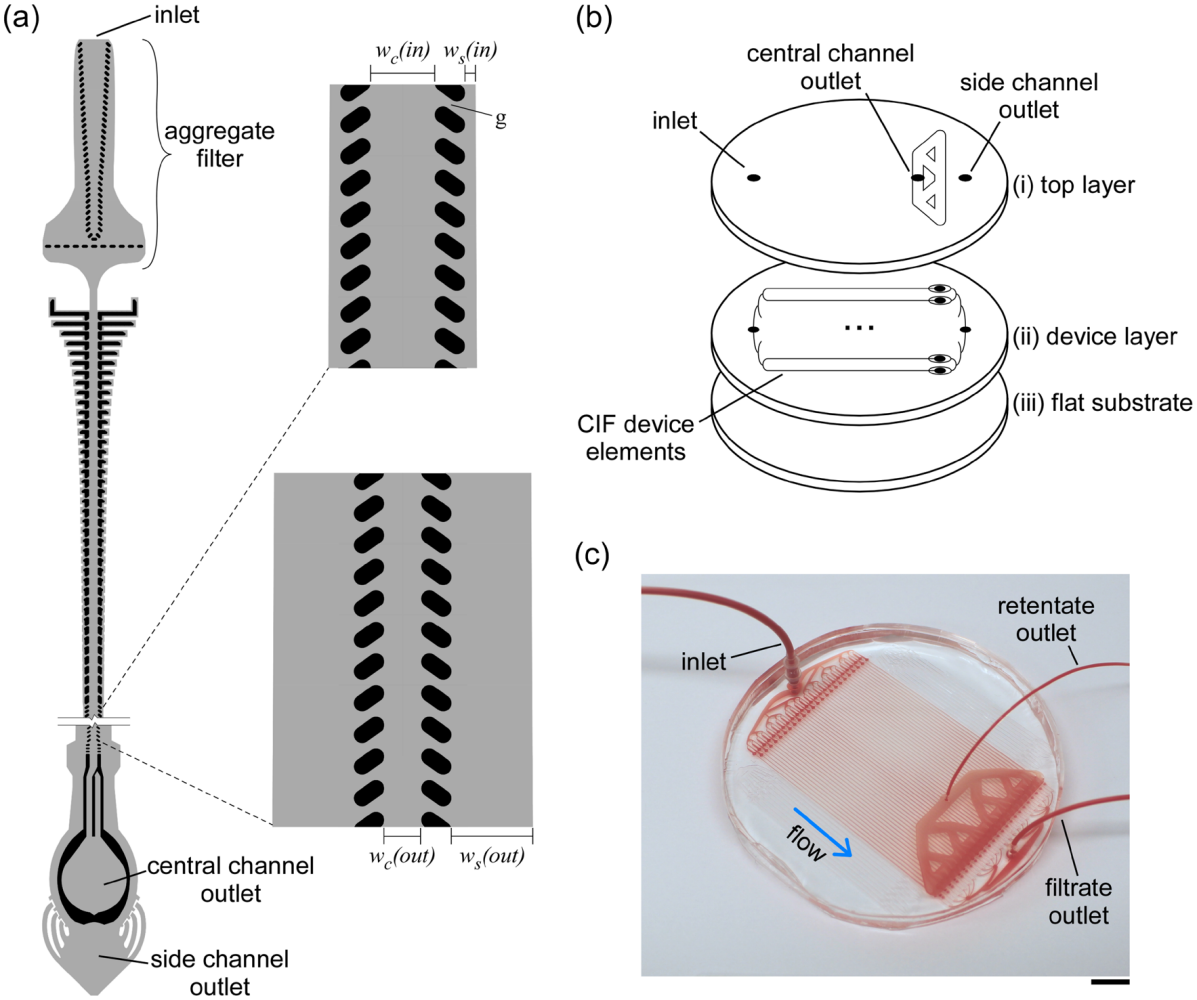
CIF-based microfluidic device. (**a**) Design of the CIF element. Width of the side channel near inlet *w*_*s*_*(in)* = 21 µm and near outlet *w*_*s*_*(out)* = 154 µm. Width of the central channel near inlet *w*_*c*_*(in)* = 120 µm and near outlet *w*_*c*_*(out)* = 70 µm. Filtration gap width *g* = 19 µm. Depth of all channels is 140 µm throughout. (**b**) Components of the multiplexed CIF device: (i) a ‘top layer’ containing a system of larger channels for collecting the central channel output (retentate) from each CIF element of the device, (ii) a ‘device layer’ consisting of individual CIF elements arranged in parallel, and (iii) a flat substrate for sealing the channels of the device. (**c**) An assembled microfluidic device comprised of 48 multiplexed CIF elements filled with a 10% HCT blood sample. Scale bar is 1 cm.

The CIF device comprised an array of 48 individual CIF elements multiplexed in parallel (Fig. 2). Each CIF element had an overall footprint of 1.4 mm × 73 mm and consisted of (Fig. 2a): (i) a built-in filter for retaining microaggregates and other debris that may be present in the blood sample, (ii) a transition region where each side channel comprised a series of progressively shorter serpentine segments to ensure the appropriate flow fraction was extracted while maintaining minimal feature size at ~ 20 µm^44^, and (iii) a linear separation region (~ 61 mm-long) in which the width of the side channels progressively increased and the width of the central channel decreased throughout its length. A series of through holes in the device layer provided fluidic access to the common inlet and the outlets of all CIF elements. The outputs of the central channels were collected through a network of large channels in the separate top layer (Fig. 2b). A fully assembled CIF device had one inlet through which the blood sample was distributed to each CIF element of the device, and two outlets for collecting the outputs of the central channels (retentate) and side channels (filtrate) of all CIF elements (Fig. 2c). The void volume of the multiplexed CIF device was 0.4 mL, excluding the tubing (Fig. 2c).

### Device separation performance in the flow-through regime

**Figure 3:**
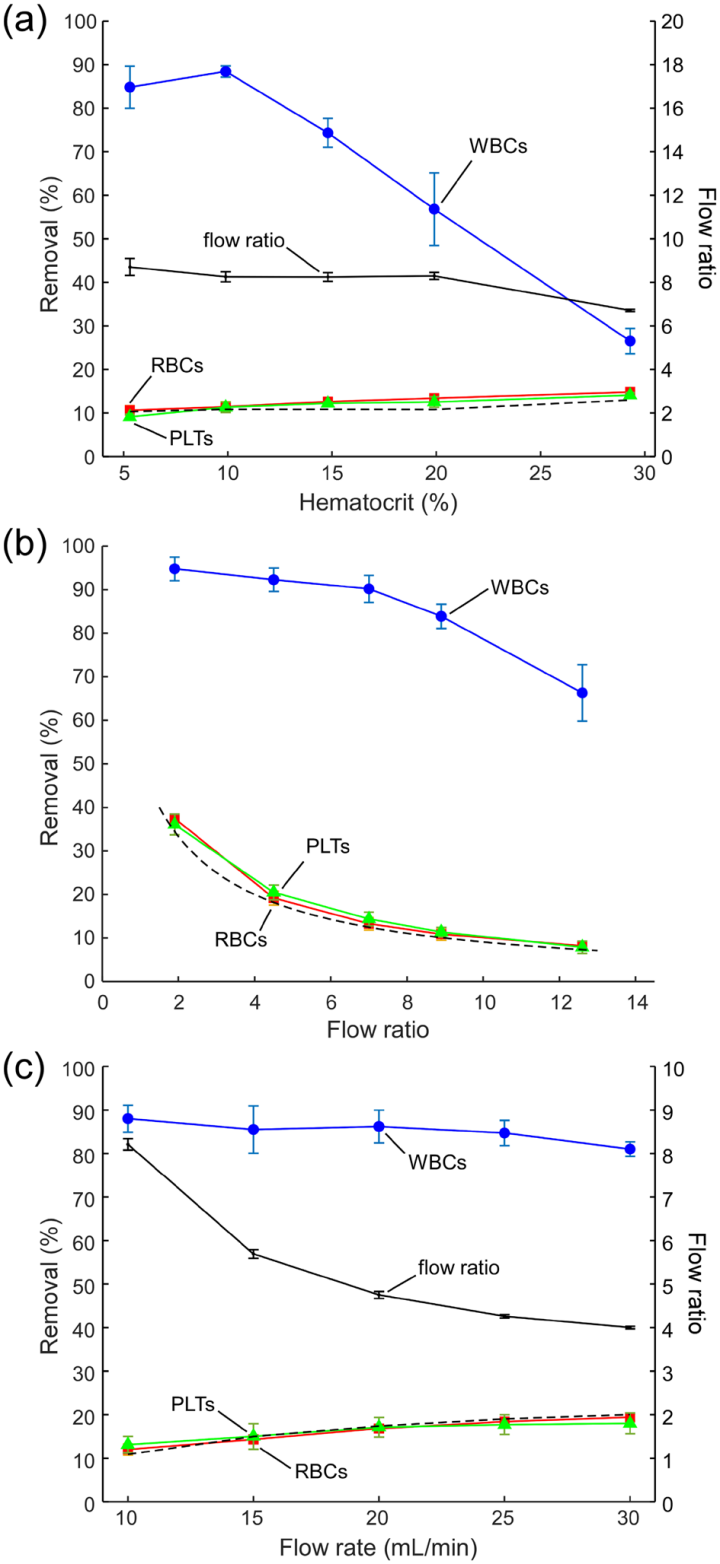
Performance of the CIF device in the flow-through regime. Dependence of cell removal on (**a**) sample HCT (10 mL/min flow rate, n = 3), (**b**) device flow ratio (10% HCT, 10 mL/min flow rate, n = 5), and (**c**) flow rate (10% HCT, n = 3). In panels (**a**) and (**c**), the flow ratio was set at 8.70 ± 0.39 and 8.21 ± 0.13 respectively, and then allowed to change depending on the parameters of the experiment. In each panel, a dashed line indicates the expected percent removal for cells that are smaller than the critical diameter of the device calculated based on the flow ratio measured in each experiment. Values shown are mean ± standard deviation.

We first tested the effect of sample HCT on the CIF device performance. The efficiency of WBC removal was the highest (88.4 ± 1.3%) for the sample with 10% HCT and decreased as the HCT of the sample increased (Fig. 3a). For samples with 20% HCT, the CIF device was able to remove 57 ± 7% of the WBCs initially present in the blood passing through the device. The percent removal (loss) of RBCs and PLTs increased modestly with increasing HCT, from 10.6 ± 0.9% for RBCs and 9.1 ± 0.5% for PLTs at 5% HCT to 14.8 ± 0.1% for RBCs and 14.1 ± 0.5% for PLTs at 30% HCT (Fig. 3a). As expected for cells that are smaller than the critical diameter of the device, RBC and PLT percent losses closely matched the values predicted based on the device flow ratio, i.e. 100/(1 + flow ratio) (Fig. 3a, dashed line).

A CIF device is designed to have a specific flow ratio, assuming the same pressure in the device outlets. To control the flow ratio in real-time, we created a difference in pressure between the outlets by varying the relative heights of the reservoirs collecting the retentate and filtrate. The actual flow ratio at which the device operated was then calculated by simply dividing the volume of the filtrate by the volume of the retentate collected from the device per unit of time. This simple manipulation allowed us to adjust the flow ratio within a wide range (~ sixfold, Fig. 3b). Lower flow ratios were associated with higher WBC removal, which remained consistently above 80% for all flow ratios up to ~ 9 (reaching 84 ± 3% at the flow ratio of 8.9 ± 1.5). The removal (loss) of RBCs and PLTs followed the expected reciprocal dependence on flow ratio (Fig. 3b, dashed line), decreasing rapidly from 37.5 ± 0.8% for RBCs and 36.1 ± 2.4% for PLTs at the flow ratio of 1.9 ± 0.1 (34% expected loss) to 10.9 ± 1.4% for RBCs and 11.4 ± 1.1% for PLTs at the flow ratio of 8.9 ± 1.5 (10% expected loss), and further down to 8.2 ± 0.9% for RBCs and 7.9 ± 1.4% for PLTs at the flow ratio of 12.6 ± 2.1 (7% expected loss) (Fig. 3b). Given such a strong dependence of RBC and PLT loss on device flow ratio, the ability to adjust the flow ratio in real-time could be useful for matching key parameters of the leukapheresis procedure (i.e., WBC removal, RBC and PLT loss) to the unique needs of individual patients.

We further evaluated the separation efficiency of the CIF device at flow rates ranging from 10 to 30 mL/min (Fig. 3c). WBC removal declined gradually with increasing flow rate from 88.0 ± 2.5% at 10 mL/min to 81.0 ± 1.4% at 30 mL/min. The flow ratio of the device also declined from 8.21 ± 0.13 at 10 mL/min down to 4.00 ± 0.03 at 30 mL/min (Fig. 3c). This decline was caused by the distortion of the CIF channel geometry (bulging) due to the elastic deformation of PDMS at higher driving pressures / flow rates, as we have previously observed for similar devices^46^. Consequently, the RBC and PLT loss increased from 12.0 ± 1.1% for RBCs and 13.1 ± 1.5% for PLTs at 10 mL/min to 19.4 ± 0.6% for RBCs and 18.0 ± 2.0% for PLTs at 30 mL/min, following the trend expected based on the flow ratio (Fig. 3c, dashed line).

### Device separation performance in the recirculation regime

**Figure 4:**
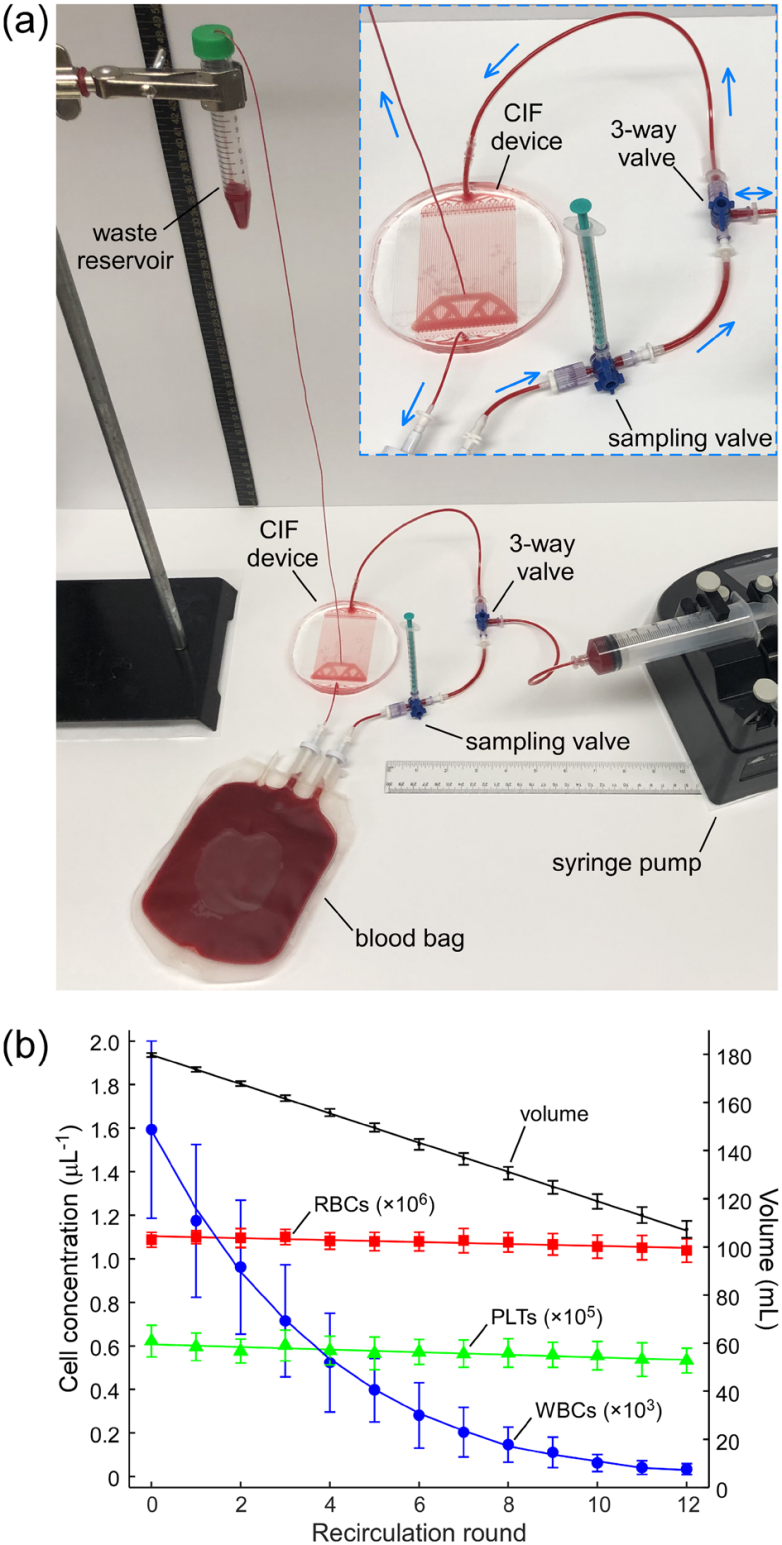
Removal of WBCs from blood during recirculation. (**a**) Experimental setup used for the recirculation experiments. The blood bag was filled with 179.7 ± 0.8 mL of WB diluted to 10% HCT with normal saline. The waste reservoir collecting the retentate (concentrated WBCs) was placed 55 cm above the device; the resulting device flow ratio was 8.8 ± 0.3. At each recirculation round, 56.5 ± 0.8 mL of the sample was withdrawn from the bag and then infused through the CIF device at a flow rate of 10 mL/min, while producing 5.6 ± 0.5 mL of retentate (waste). A 1-mL syringe was used to sample the blood coming from the bag. Arrows indicate the direction of flow of blood in the circuit. (**b**) Changes in cell concentrations following each round of CIF-based cell separation in the recirculation regime. Values shown are mean ± standard deviation (n = 9, using blood from 6 unique subjects). Solid lines are linear fits for volume (y = − 0.0608x + 1.7975, R^2^ = 0.9997), RBCs (y = − 0.0044x + 1.1037, R^2^ = 0.8218) and PLTs (y = − 0.0059x + 0.6072, R^2^ = 0.8485), and a model fit for WBCs (with WBC removal of 81% minimizing the root-mean-square error).

Next, we tested the CIF device in the recirculation regime using the operational parameters (flow rate, flow ratio, sample HCT) that maximized device performance in the flow-through experiments. In our recirculation setup (Fig. 4a), a blood bag filled with 179.7 ± 0.8 mL of diluted WB (10% HCT) was used to emulate the TBV of a subject. During each recirculation round, 56.5 ± 0.8 mL of the blood sample was withdrawn from the bag (via a length of tubing inserted all the way to the bottom of the bag through one of its sampling ports) and then passed through the CIF device at a flow rate of 10 mL/min. The retentate (5.6 ± 0.5 mL per round) was collected into a conical tube, which was elevated above the CIF device to produce the desired flow ratio of 8.8 ± 0.3 (Fig. 4a). To calculate the flow ratio for a device operating in the recirculation regime, the volume of the filtrate was estimated as the difference between the volume pushed through the device and the volume of the retentate collected into the waste reservoir per recirculation round. The filtrate was returned to the bag through the other sampling port at the top of the bag, to effectively place the blood ‘inlet’ and ‘outlet’ at the opposite sides of the bag and thus promote mixing. Additionally, the blood bag was mixed during and after each recirculation round to ensure a uniform distribution of cells within the bag. This ‘withdraw-infuse’ cycle was repeated 12 times over ~ 3.5 hours. Following the completion of each round, a 0.2 mL sample was taken from the bag (via the sampling valve) and the retentate for measurements. The volumes of the retentate and each sample withdrawn were recorded to track the change in volume of the blood bag after each recirculation round.

The concentration of WBCs in the blood bag declined exponentially throughout the recirculation experiment (Fig. 4b). After 3 recirculation rounds (~ 170 mL processed volume), WBC concentration decreased by 55%, from 1.59 ± 0.41 × 10^3^/µL in the initial sample down to 0.72 ± 0.26 × 10^3^/µL. At round 6 (~ 340 mL processed volume), WBC concentration decreased further down to 0.28 ± 0.15 × 10^3^/µL (82% decrease from the initial level). By the end of the recirculation experiment (round 12, ~ 680 mL processed volume), WBCs in the bag were virtually depleted (~ 98% decrease from the initial level). The concentration of RBCs and PLTs decreased linearly over the entire duration of the recirculation experiment (Fig. 4b): by ~ 4.5% for RBCs (from 1.09 ± 0.03 × 10^6^/µL to 1.04 ± 0.05 × 10^6^/µL; y = − 0.0044x + 1.1037, R^2^ = 0.8218), and by ~ 14.5% for PLTs (from 0.62 ± 0.07 × 10^5^/µL to 0.53 ± 0.06 × 10^5^/µL; y = − 0.0059x + 0.6072, R^2^ = 0.8485). Cells that are smaller than the critical diameter of the CIF device distribute according to the flow ratio, and therefore their concentration should be the same in both filtrate and retentate. Hence, we investigated the effect of blood recirculation through the CIF device on the properties of blood cells.

### Effect of device operation in recirculation regime on blood cell properties

**Figure 5:**
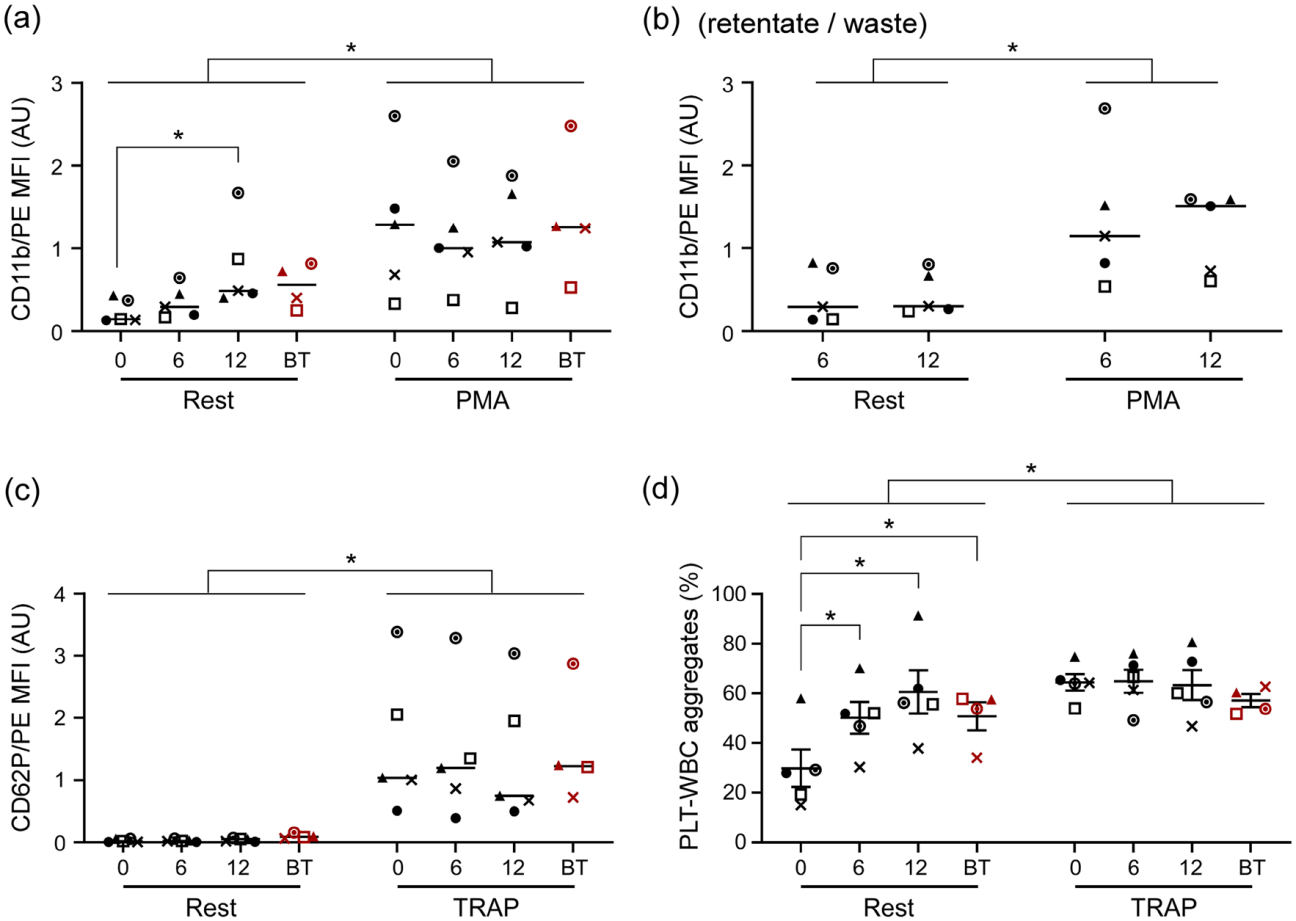
Effect of CIF processing in the recirculation regime on the activation state of WBCs and PLTs. (**a**) Activation of WBCs (CD11b) remaining in the recirculating blood. (**b**) Activation of WBCs (CD11b) removed with the retentate (waste). (**c**) Activation of recirculating PLTs (CD62P). (**c**) Formation of PLT-WBC aggregates in the recirculating blood. Measurements were performed via flow cytometry for samples collected before (round 0), during (round 6), and after (round 12) the recirculation experiment, both at rest and after additional activation with PMA (panels a & b) or TRAP (panels c & d). BT (red symbols) represent blood samples set aside for the duration of each recirculation experiment to assess the effect of time alone on cell activation. Statistical significance is denoted by * for *p* < 0.05. Each symbol represents a different recirculation experiment (n = 5, using blood from 4 unique subjects).

We used imaging flow cytometry (FC) to measure activation of WBCs (CD11b) and PLTs (CD62P), and PLT-WBC aggregate formation for samples collected from the bag before (round 0), during (round 6), and immediately after (round 12) the recirculation experiments (Fig. 5). Activation of separated WBCs (retentate output) was measured during (round 6) and after (round 12) the experiment. Additionally, in four out of five recirculation experiments, a sample of the subjects’ blood was set aside on the benchtop (BT) for the duration of the experiment to control for activation of WBCs and PLTs from simply being stored at room temperature for a prolonged period. All samples were tested at rest (as collected) and after incubation with either phorbol 12-myristate 13-acetate (PMA; for WBCs, Fig. 5a, b) or thrombin receptor agonist peptide-6 (TRAP; for PLTs and PLT-WBC aggregates, Fig. 5c, d) to evaluate how CIF recirculation affected the ability of WBCs and PLTs to become activated in response to relevant stimuli.

WBCs in recirculating blood become progressively more activated, but at the end of the recirculation experiment (after round 12), they were no more activated than the BT control (Fig. 5a). WBCs removed with the retentate were about as activated as WBCs remaining in the recirculating blood, and there was no significant difference in activation between cells extracted at rounds 6 and 12 (Fig. 5b). Both recirculating and removed WBCs were able to become significantly more activated after incubation with PMA (positive control) regardless of when they were sampled during the recirculation experiment, which suggests that the cells did not become refractory after processing (Fig. 5a, b).

Despite the potential effect of shear on PLTs, activation of PLTs in the recirculating blood did not increase significantly over the entire duration of the experiment. After round 12 of recirculation through the CIF device, the level of PLT activation was similar to that of the BT control (Fig. 5c). Low activation could be caused by PLT refractoriness, which was not the case because recirculating PLTs were able to activate significantly upon exposure to TRAP (positive control) (Fig. 5c). Given the high sensitivity of PLTs to shear and our previously published findings with similar CIF devices^43–45^ we expected to observe at least some increase in activation of recirculating PLTs. One possible explanation is that most of the PLTs that became activated got promptly bound to the available WBCs to form PLT-WBC aggregates. Indeed, the number of PLT-WBC aggregates increased steadily and significantly over time during each recirculation experiment (Fig. 5d). PLTs participating in the PLT-WBC aggregates are excluded when PLT activation and count are measured, which can explain the activation of recirculating PLTs remaining relatively low (Fig. 5c) and their concentration gradually declining over time (Fig. 4b). Overall, the number of PLT-WBC aggregates in the recirculating blood after 12 rounds of recirculation was the same as in the BT control (Fig. 5d), suggesting that the increase was likely due to processing duration, and contribution from the CIF device was relatively minor. It further follows that one potential solution for minimizing the cell activation and PLT-WBC aggregate formation would be to reduce processing duration by increasing either the volumetric throughput (e.g., via additional multiplexing) or the separation efficiency (e.g., via additional design improvements) of the device.

Finally, we tested whether recirculation through the CIF device damaged RBCs by measuring free hemoglobin (Hb) and potassium levels before (round 0), during (round 6), and immediately after (round 12) each experiment. We previously employed the same assays to sensitively identify sublethal damage caused by centrifugation to stored RBCs during washing^47,48^. Throughout the CIF recirculation procedure, we did not observe any significant changes in free Hb levels and potassium levels remained below the detectable level (< 0.2 mM) in all samples (Table S1). Absent any indication of hemolysis, the most likely explanation for the observed decline of RBC concentration in the recirculating blood (Fig. 4b) is that an excessive number of RBCs was removed with the retentate. Indeed, the RBC concentration in the retentate (waste) was consistently higher than in the recirculating blood (Fig. 4b), on average by about 0.1 × 10^6^/µL (Table S2). As discussed in our earlier study in which we observed a similar buildup of RBCs in the retentate when processing leukapheresis samples^46^, RBCs are highly deformable biconcave discs (~ 8 µm in diameter and only ~ 2 µm thick) and therefore their effective diameter could vary widely depending on the specific conditions near each filtration gap. Most RBCs have an effective diameter sufficiently small to follow the filtrate, but some do not and therefore tend to remain in the central channel creating the characteristic buildup^46^.

## Discussion

Conventional apheresis machines utilize centrifugation to separate WBCs from RBCs and PLTs, which limits the degree to which the ECV of the leukapheresis circuit could be reduced to match the needs of pediatric patients. The void volume of the CIF device (0.4 mL) described in this study was at least 100-fold smaller than that of a typical centrifugation-based apheresis machine (150–250 mL)^49^. This dramatic reduction of the ECV represents the main advantage of using microfluidic cell separation to ultimately enable safe and effective leukapheresis procedure in young children. This study builds upon our earlier CIF designs^46^, to tackle an entirely novel, much more challenging application than what we have explored previously. For the first time, a CIF microfluidic device is demonstrated capable of separating WBCs from diluted whole blood with > 80% efficiency while operating in the recirculation regime for > 3 hours without a noticeable decline in separation performance and with minimal effect on cell properties. Additionally, the current device operated optimally at an HCT that was 2 times higher, and with RBC and PLT loss that was 2–3 times lower, than in any of our previous reports^46^.

Pediatric patients undergoing leukapheresis for leukodepletion or cellular collection are typically anemic, with an average HCT of 20–30%^49,50^. At 20% HCT, the CIF device had a WBC removal efficiency of ~ 60%, which may be an acceptable level of performance in some patients. To operate the current CIF device prototype at its peak separation efficiency (WBC removal > 80%), blood would have to be diluted to 10% HCT before entering the device and then concentrated back to its native HCT before returning to the patient. Such hemoconcentration is performed routinely during pediatric cardiopulmonary bypass and extracorporeal membrane oxygenation to remove excess fluids, using devices with excellent biocompatibility and minimal void volume (e.g., 8 mL, Hemocor HPH Junior, Minntech Corp., Minneapolis, MN) at flow rates compatible with leukapheresis (as high as 100 mL/min)^51^. The decline in separation performance of the device at higher HCTs could be explained by the increasing number of stochastic cell–cell interactions and the deviation of the apparent viscosity of blood in the channels from the model we used when designing the device, both of which become more pronounced with increasing HCT^46,52,53^. Additional research to address these factors will be needed to further increase the CIF separation efficiency at higher HCTs.

The flow rate at which the CIF device operated with peak separation performance in our study (10 mL/min) is similar to clinical leukapheresis procedures, which are typically performed at 10–50 mL/min^1,17,54^. The observed decline in the separation efficiency at higher flow rates was likely due to the deformation (bulging) of the device channels at higher driving pressures, as we previously observed for similar devices^46^. This effect could be minimized by ultimately fabricating CIF devices from hard thermoplastics rather than the PDMS elastomer we used in this study^39,40,55^.

The HCT (10%) and flow rate (10 mL/min) at which the CIF device operated with its optimal performance were significantly higher than previously reported for other microfluidic devices designed to separate WBCs from minimally diluted whole blood^39,40,42,56–61^. Importantly, this study was the first to demonstrate a microfluidic device capable of highly efficient separation of cells from blood while operating in the recirculation regime, as opposed to the flow-through regime employed by all other microfluidic cell separation devices^32^.

The recirculation in this study was accomplished using a syringe pump programmed to go through multiple ‘withdraw-infuse’ cycles over the entire duration of the procedure. Such an approach to recirculation closely resembles the operation of an apheresis machine in the discontinuous mode typically employed for larger ECV or longer duration procedures^1,49^, or the manual exchange transfusion sometimes performed for young children when centrifugation-based leukapheresis is considered too risky due to a patient’s hemodynamic status, relatively small TBV compared to the ECV of the circuit, and/or ability to place an appropriately sized catheter^28,62^. Remarkably, the CIF device was able to process blood without any signs of clogging while maintaining its separation efficiency for > 3 h, which is the typical duration of a conventional leukapheresis procedure^1,17,54,62^. This finding is particularly significant because any device intended for leukapheresis must be able to process recirculating blood over an extended period of time.

In the context of leukoreduction, conventional centrifugation-based leukapheresis would typically reduce WBC concentration by about one-third when processing 1–2 TBV^50^ and by about one-half when processing 2–3 TBV^63^. The CIF device was able to reduce the WBC concentration in the recirculating blood about twice as fast—by one-half after processing just one TBV, and by four-fifths after processing two TBV. From the clinical perspective, minimizing the loss of RBCs and PLTs during the leukapheresis procedure is important for reducing the risks associated with transfusion of allogeneic blood products. The loss of PLTs during the CIF device operation was ~ 2–3 times lower than would be typical for a centrifugation-based procedure (for which PLTs are removed at the same rate as WBCs because both cell types co-localize in the ‘buffy coat’ layer)^50,63^. Additional research will be needed to increase the flow ratio of the CIF device to further reduce the RBC and PLT losses.

Exposure to high shear forces during centrifugation can cause mechanical damage to cell membranes, induce excessive cell activation, and trigger hemostatic responses, contributing to the host of adverse outcomes associated with leukapheresis in neonates and infants^21,24,28,64^. In our experiments, WBCs and PLTs in the recirculating blood were no more activated than the cells in the BT control (sample left on the bench for the duration of the procedure), suggesting minimal additional activation contributed by the CIF device. Additionally, both WBCs and PLTs were able to become activated after exposure to an appropriate stimulant, suggesting that the cells were not refractory after CIF processing. Finally, we found no evidence of damage to the RBCs either. Taken together, our data strongly suggest that the CIF device operating in the closed-loop recirculation regime did not significantly activate or damage the blood cells.

Centrifugation is used to perform leukapheresis because it has been the only technology capable of separating WBCs with the required efficiency and volumetric throughput from recirculating blood. This study demonstrates the feasibility of using high-throughput microfluidic cell separation technology to ultimately enable centrifugation-free, low-ECV leukapheresis. Such a capability would be particularly useful in young children, a vulnerable group of patients who are currently underserved.

## Materials and methods

### Blood samples

All experiments were performed in accordance with guidelines and regulations established by the University of Houston and the U.S. Department of Health and Human Services for the protection of human subjects. All experimental protocols involving human blood samples were approved by the University of Houston Institutional Review Board (Committee for the Protection of Human Subjects 1, protocol #16272-01). Informed consent was obtained from all subjects and/or their legal guardian(s). Units of whole blood (WB) were purchased from the Gulf Coast Regional Blood Center (Houston, TX). Samples of fresh WB were obtained by venipuncture from healthy volunteers (anticoagulant: acid citric dextrose, solution A; Vacutainer, BD Biosciences, Franklin Lakes, NJ). Samples were used immediately or stored in a blood bank refrigerator (4 °C, iB111, Helmer Scientific, Noblesville, IN) until use, and diluted with isotonic saline (0.9% w/v NaCl, RICCA Chemical Company, Arlington, TX) to achieve the desired hematocrit (HCT).

### Device fabrication

The design and fabrication of devices based on controlled incremental filtration (CIF) technology have been previously described in detail^43–46^. In a typical CIF design, the width of all filtration gaps is fixed at ~ 20 µm because microfluidic devices with a ‘minimum feature size’ less than 20 µm are very difficult to mass produce using currently available manufacturing methods^43^. Similarly, manufacturability of microfluidic devices with aspect ratio (depth:width) above ~ 7:1 is very limited. Therefore, CIF devices are designed to have channel depths of ~ 140 µm to have fluidic resistance as low as possible (and therefore higher flow rate/throughput at a given driving pressure) while still maintaining the fundamental manufacturability of the design. Given that the width of the gaps is fixed, the fraction of flow passing from the central to the side channels at each gap is controlled by the change in the widths of the channels, as described previously in detail^44^. If this filtration flow fraction is zero, no fluid is flowing through the gaps, and therefore no cells are carried by the fluid into the side channels. As the filtration flow fraction increases, the amount of fluid flowing through each gap increases, and therefore the size of the cells that are sufficiently small to be pulled by the flow into the side channels also increases. Cells that are too large to be pulled by the fluid flowing through the gaps will remain in the central channel. Thus, the magnitude of the fluid flow through the gaps determines the separation size cutoff (not the gap width which remains constant). This decoupling of the size cutoff from the gap width allows CIF devices with 20 µm gaps to successfully separate particles/cells that are smaller than the gaps, as demonstrated previously^43–46^.

The CIF device design, generated using custom code in MATLAB (The MathWorks Inc, Natick, MA), was transferred into a ~ 140 µm-thick layer of photoresist (SU-8 3050; Kayaku Advanced Materials Inc, Westborough, MA) on a 4″ silicon wafer (University Wafer, South Boston, MA) using soft lithography. The master wafer was replicated in poly(dimethylsiloxane) (PDMS; Sylgard 184, Dow Corning Corp, Midland, MI), and the PDMS replica (device layer) was sealed against a PDMS-coated Petri dish (flat substrate) using oxygen plasma (PDC-001, Harrick Plasma, Ithaca, NY). The inlet and outlet ports in the device layer were created using biopsy punches (Acuderm Inc, Fort Lauderdale, FL) of the appropriate size to match tubing connections (1.02- and 0.58-mm inner diameters; Scientific Commodities, Havasu City, AZ). An additional PDMS layer containing a system of large channels for collecting the filtrate and retentate outputs from the individual CIF elements of a multiplexed device was bonded on top of the device layer. After bonding, each assembled CIF device was treated with 1% (w/v) aqueous solution of mPEG-silane (MW 5000, Laysan Bio Inc, Arab, AL) for 25 min at 70 °C. Finally, the device was flushed with GASP buffer (9 mM Na_2_HPO_4_, 1.3 mM NaH_2_PO_4_, 140 mM NaCl, 5.5 mM glucose, 1% w/v bovine serum albumin, 290 mmol/kg, pH 7.4) and stored at 4 °C until use.

### Recirculation setup

The recirculation setup consisted of a blood bag (500 mL; Fenwal 4R1590, GenesisBPS, Ramsey, NJ) which was connected to the multiplexed CIF device and other components of the circuit by plastic tubing (Scientific Commodities) linked through appropriately sized Luer-lock connectors (Qosina, Ronkonkoma, NY). The tubing conveying the blood sample from the bottom of the blood bag to the CIF device was connected to a syringe pump (Genie Touch, Kent Scientific, Torrington, CT) and the inlet of the CIF device via two 3-way stopcocks (Qosina). One stopcock was used for sampling the blood coming from the bag, and the other stopcock was used for connecting the syringe pump with either the outlet of the bag (to withdraw blood from the bag) or the inlet of the device (to infuse blood through the device). The mode of pump operation (infuse/withdraw) and the position of the stopcock were set manually during the experiment. The blood bag was mixed by hand during and after each recirculation round.

### Measurements of separation performance and cell properties

Complete blood counts with a 5-part differential were measured using a hematology analyzer (XS-1000i, Sysmex America, Inc., Mundelein, IL). WBC, RBC and PLT counts were used to calculate the percent removal for each cell type as follows: $\% removal={C}_{r}/\left({C}_{r}+{C}_{f}\times FR\right)\times 100$, where ${C}_{r}$ is the cell count in the retentate (central channel output of all CIF elements), ${C}_{f}$ is the cell count in the filtrate (side channel output of all CIF elements), and $FR$ is the flow ratio of the device (defined as the ratio of the cumulative volume of the filtrate output to that of the retentate output of the device)^46^. All flow ratios were calculated based on measured volumes.

Imaging flow cytometry (FC, Amnis Imagestream^X^ Mk II, Luminex Corporation, Austin, TX) was used to measure PLT and WBC surface antigen activation markers, and the prevalence of PLT-WBC aggregates, using the following antibody cocktails (all from BD Biosciences, San Jose, CA). PLT activation: CD41a/APC (20 µL; BD 559,777), CD62P/PE (20 µL; BD 555,524), Dulbecco’s phosphate-buffered saline without calcium or magnesium (DPBS-/-; 10 µL). Thrombin receptor agonist peptide 6 (TRAP; 70 µM; Sigma) was used as the positive control for PLT activation. WBC activation: CD45/APC (5 µL; BD 561,864), CD62L/FITC (20 µL; BD 555,543), CD11b/PE (20 µL; BD 555,388), and DPBS-/- (25 µL). Phorbol myristate acetate (PMA; 0.5 µg/mL; Sigma) was used as the positive control for WBC activation. PLT-WBC aggregates (PLA): CD45/APC (5 µL), CD41a/FITC (20 µL; BD 555,466), and DPBS-/- (45 µL). TRAP (70 µM) was used as the positive control for PLA formation. To perform the FC measurements, 30 µL of the blood sample were added to each antibody cocktail, gently mixed, and left to incubate in the dark for 15 min at room temperature (RT). After antibody labeling, RBCs were lysed using 1X BD FACS lysing solution for 15 min at RT. After lysis, samples were centrifuged at 900 × g for 5 min to remove the supernatant, pelleted cells were resuspended in 100 µL of 1% paraformaldehyde and then stored at 4ºC until FC measurements were performed (within 24 h).

The level of free hemoglobin (Hb) in the supernatant was measured using the modified cyanmethemoglobin method following the manufacturer’s instructions (Drabkin’s reagent; D5941, Sigma). Briefly, a blood sample was centrifuged at 1000 × g for 5 min to pellet the RBCs, then 40 µL of the supernatant was added to 160 µL of Drabkin’s reagent, and incubated for 20 min. The absorbance was measured at 540 nm using a plate reader (SpectraMax M5, Molecular Devices, Sunnyvale, CA). The concentration of Hb was calculated using a calibration curve constructed with a human Hb standard (Pointe Scientific Inc, Canton MI). The level of potassium (K^+^) was measured using a handheld blood analyzer (i-STAT, Abbott Laboratories, Abbott Park, IL) using CHEM8 + cartridges, as previously described^47^.

### Statistical analysis

All values were expressed as mean ± standard deviation. Statistical significance (defined as *p* < 0.05) of the observed differences was determined using the paired two-sided t-test for cell count data, one-way ANOVA for Hb measurements, and either 2-way repeated measures ANOVA or a mixed-effects model (restricted maximum likelihood) matched for both time and activation state with Sidak’s multiple comparisons test for FC measurements.

## Supplementary Information

Supplementary Information.
