# Supplementary material for: A high-throughput microfluidic device based on controlled incremental filtration to enable centrifugation-free, low extracorporeal volume leukapheresis
Source: Sci Rep. 2022 Aug 13;12:13798. doi: 10.1038/s41598-022-16748-5 (PMC9376077; doi:10.1038/s41598-022-16748-5)
Supplement: Supplementary file 1 — Supplementary Information. [file 41598_2022_16748_MOESM1_ESM.docx]

**Supplementary Information**

**A high-throughput microfluidic device based on controlled incremental filtration to enable centrifugation-free, low extracorporeal volume leukapheresis**

Dalia L. Lezzar,^1^ Fong W. Lam,^2^ Ravin Huerta,^1^ Anton Mukhamedshin,^1^ Madeleine Lu,^1^
Sergey S. Shevkoplyas^1,*^

**Table S1.** Free hemoglobin (Hb) and potassium (K+) measurements (mean of triplicate) taken before, during, and immediately after the 12 consecutive rounds of recirculation through the device (n = 5, using blood from 4 unique subjects); and measurements taken from a control blood sample left to sit in a tube on the lab bench at room temperature (n = 4 using blood from 4 unique subjects). Values shown are mean ± standard deviation.

|  | **Recirculation Trial** | **1** | **2** | **3** | **4** | **5** |
| --- | --- | --- | --- | --- | --- | --- |
| **BEFORE (Round 0)** | **Free Hb (g/dL)** | 0.2253 ± 0.003 | 0.2866 ± 0.0001 | 0.3419 ± 0.002 | 0.3170 ± 0.001 | 0.3550 ± 0.001 |
|  | **K+ (mmol/L)** | < 2.0 | < 2.0 | < 2.0 | < 2.0 | < 2.0 |
| **DURING (Round 6)** | **Free Hb (g/dL)** | 0.2272 ± 0.001 | 0.2881± 0.003 | 0.3445 ± 0.001 | 0.3147 ± 0.002 | 0.3572 ± 0.004 |
|  | **K+ (mmol/L)** | < 2.0 | < 2.0 | < 2.0 | < 2.0 | < 2.0 |
| **AFTER (Round 12)** | **Free Hb (g/dL)** | 0.2315 ± 0.009 | 0.2870 ± 0.0004 | 0.3445 ± 0.002 | 0.3163 ± 0.005 | 0.3599 ± 0.005 |
|  | **K+ (mmol/L)** | < 2.0 | < 2.0 | < 2.0 | < 2.0 | < 2.0 |
| **BENCH CONTROL (after Round 12)** | **Free Hb (g/dL)** | n/a | 0.2894 ± 0.001 | 0.3462 ± 0.003 | 0.3140 ± 0.001 | 0.3586 ± 0.001 |
|  | **K+ (mmol/L)** | n/a | < 2.0 | < 2.0 | < 2.0 | < 2.0 |

**Table S2** Difference in RBC concentration between the retentate and filtrate of the CIF device operating in the recirculation regime (n = 9, using blood from 6 unique subjects).

| **Recirculation round** | **Mean difference, ×10^6^/µL** | **Standard deviation, ×10^6^/µL** |
| --- | --- | --- |
| 1 | 0.03 | 0.13 |
| 2 | 0.06 | 0.15 |
| 3 | 0.07 | 0.12 |
| 4 | 0.11 | 0.11 |
| 5 | 0.12 | 0.11 |
| 6 | 0.13 | 0.11 |
| 7 | 0.13 | 0.12 |
| 8 | 0.12 | 0.11 |
| 9 | 0.13 | 0.10 |
| 10 | 0.13 | 0.10 |
| 11 | 0.13 | 0.12 |
| 12 | 0.12 | 0.11 |
